# Susceptibility of different leukocyte cell types to Vaccinia virus infection

**DOI:** 10.1186/1743-422X-1-10

**Published:** 2004-11-22

**Authors:** Juana M Sánchez-Puig, Laura Sánchez, Garbiñe Roy, Rafael Blasco

**Affiliations:** 1 Departamento de Biotecnología-I.N.I.A. Ctra. La Coruña km 7.5 E-28040 Spain; 2 Servicio de Inmunología. Hospital Ramón y Cajal. 28034 Madrid, Spain

## Abstract

**Background:**

Vaccinia virus, the prototype member of the family *Poxviridae*, was used extensively in the past as the Smallpox vaccine, and is currently considered as a candidate vector for new recombinant vaccines. Vaccinia virus has a wide host range, and is known to infect cultures of a variety of cell lines of mammalian origin. However, little is known about the virus tropism in human leukocyte populations. We report here that various cell types within leukocyte populations have widely different susceptibility to infection with vaccinia virus.

**Results:**

We have investigated the ability of vaccinia virus to infect human PBLs by using virus recombinants expressing green fluorescent protein (GFP), and monoclonal antibodies specific for PBL subpopulations. Flow cytometry allowed the identification of infected cells within the PBL mixture 1–5 hours after infection. Antibody labeling revealed that different cell populations had very different infection rates. Monocytes showed the highest percentage of infected cells, followed by B lymphocytes and NK cells. In contrast to those cell types, the rate of infection of T lymphocytes was low. Comparison of vaccinia virus strains WR and MVA showed that both strains infected efficiently the monocyte population, although producing different expression levels. Our results suggest that MVA was less efficient than WR in infecting NK cells and B lymphocytes. Overall, both WR and MVA consistently showed a strong preference for the infection of non-T cells.

**Conclusions:**

When infecting fresh human PBL preparations, vaccinia virus showed a strong bias towards the infection of monocytes, followed by B lymphocytes and NK cells. In contrast, very poor infection of T lymphocytes was detected. These finding may have important implications both in our understanding of poxvirus pathogenesis and in the development of improved smallpox vaccines.

## Background

Vaccinia virus, the prototype of the *Poxviridae*, is a large DNA virus whose replication takes place in the cytoplasm of the infected cell [1]. Although well characterized *in vitro*, little is known about the ability of vaccinia virus to infect different cell types *in vivo*. Vaccinia virus host range in cell culture is known to be determined by several genes. The importance of host restriction has been highlighted in recent years by the growing use of the Modified Vaccinia Ankara (MVA) virus strain, whose replication is severely impaired in human cells [2-4]. Genes known to influence the ability of vaccinia virus to infect cells, termed host range genes, have been identified, and shown to block productive infection at different points in the replication cycle. Significantly, MVA replication of non-permissive cells proceeds through early and late gene expression, but is blocked at late times in a step of virion morphogenesis [5].

In addition to host range genes, there are a number of factors that might influence the infection rate of a given cell type, such as the accessibility and amount of receptors, the ability to internalize the virus, and the metabolic state of the cell. In addition to cellular factors, genetic differences in the virus might influence the efficiency and fate of the infection. For instance, cellular nucleotide pools can be one of the factors that, in conjunction with the expression of viral thymidine kinase (TK), may influence the rate of infection.

The above considerations led us to hypothesize that, although receptors for vaccinia seem to be ubiquitous, and virus replication is relatively independent from the host cell, virus tropism *in vivo *may be determined by many complex factors that may be dependent on the cell type and metabolic state.

We have focused here on the differences between two widely used strains of vaccinia virus (Western Reserve-WR and MVA), and also to their respective TK(-) mutants, in their ability to infect different cell types in fresh human PBLs.

## Results

### Infection of human PBLs by GFP-expressing vaccinia virus

**Figure 1:**
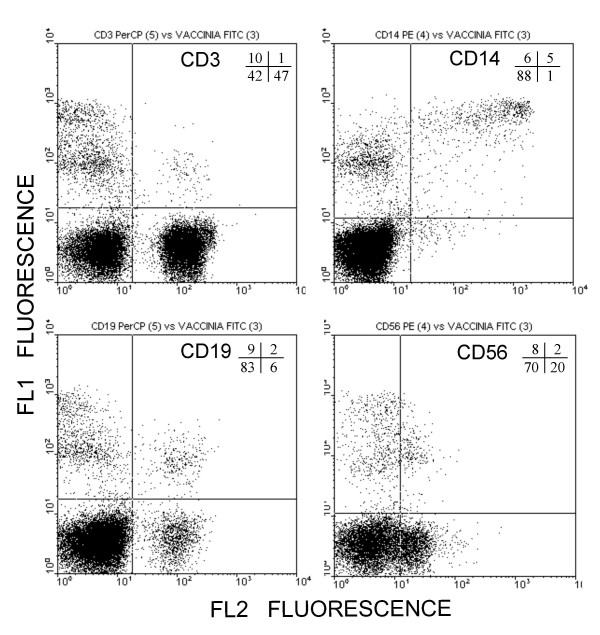
**Analysis of vaccinia infected human PBLs**. Human PBLs infected with vaccinia WR-GPF for 5 h were subsequently stained with cell-type specific mAbs, and analyzed by flow cytometry. Plots show the level of GFP fluorescence (recorded in the FL1 channel) versus the amount of labeling with the indicated antibody markers (recorded in the FL2 channel). Numbers inside the plots indicate the percentage of cells within the respective regions.

Previously, we have shown that GFP expression from a vaccinia virus recombinant can be used to monitor infection by flow cytometry [6]. Where adequate marker molecules for different cell populations exist, this approach should facilitate the study of the susceptibility of cell types to vaccinia virus infection. With this aim, fresh human PBLs from healthy donors were infected with virus WR-GFP, and analyzed by flow cytometry at different times post-infection. The overall rate of infection, measured as GFP-positive cells, was 4.5%, 7.6% and 10.0% at 1, 3 and 5 h, respectively. Staining with antibodies to CD3, CD14, CD19 and CD56 was performed on infected cells at 5 h.p.i. (Fig. 1). A marked preference was noted for the infection of non-T cells, since GFP positive cells amounted to 19% of non-T lymphocytes, while only 1.9% of T cells were infected. Among the non-T lymphocytes, there was a strong bias towards the infection of CD14 positive cells (monocytes), of which up to 77% showed green fluorescence, followed by B lymphocytes (CD19^+^, up to 20%) and NK cells (CD56^+^, up to 9%).

### Construction of MVA-GFP, WR-TK(-) and MVA-TK(-)

Vaccinia virus MVA and TK-deficient viruses have been proposed as improved recombinant vaccines. In particular, the highly attenuated MVA strain has elicited much interest as a safer vaccine vector. We studied the influence of the virus strain and the TK phenotype in the infection of human PBLs. We thus constructed GFP expressing viruses from vaccinia virus MVA strain, by inserting the GFP cassette downstream of the F13L gene, using an intergenic region for the insertion. Additionally, thymidine kinase-deficient virus recombinants WR-TK(-) and MVA-TK(-) were constructed by inserting the GFP cassette within the viral TK locus. Those viruses grew to high titers and produced, upon infection of cell lines, bright GFP fluorescence (not shown).

### Infection of human PBLs with MVA-GFP

**Figure 2:**
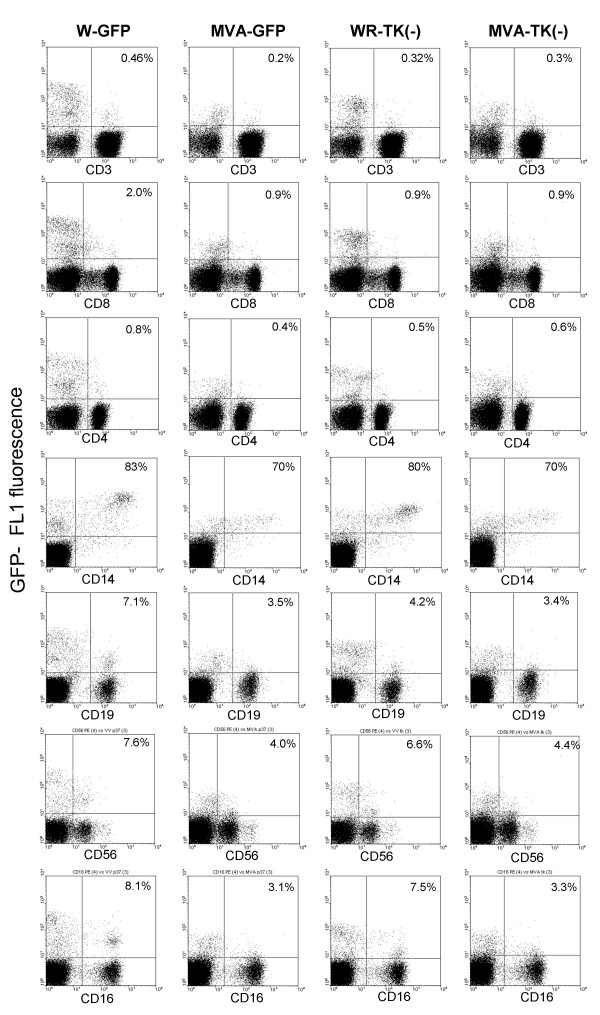
**Analysis of cell populations infected with different vaccinia viruses. **Human PBLs were infected with vaccinia virus strains WR and MVA, or their respective TK(-) mutants. PBL-subsets were identified by staining with the specific mAbs indicated under each plot. Numbers inside the plots indicate the percentage of GFP-expressing cells within each PBL-subset.

The four GFP-expressing viruses were used to infect fresh human PBLs from a different individual, and subjected to flow cytometry analysis at 7 h.p.i. (Fig. 2). The results confirmed the above findings with respect to the low infection rate of T cells in comparison with monocytes, B and NK cells. Both CD4^+ ^and CD8^+ ^cells were poorly infected, although there was indication of an increased infection of low-CD8 T lymphocytes in comparison with high-CD8 cells. Notably, this experiment confirmed that most of the monocytes (CD14+) was infected in our experimental conditions, and showed a high level of GFP fluorescence. It was of interest to directly compare the ability of vaccinia MVA to infect PBLs with that of the standard laboratory strain WR. A side-by-side comparison of WR-GFP and MVA-GFP showed that both viruses infected a high percentage of CD14^+ ^monocytes (83 and 70%, respectively), and a low percentage of T lymphocytes (0.46 and 0.2%, respectively). No significant differences were noted in the percentage of CD4 cells infected with both viruses. Although both virus strains were able to infect the majority of monocytes, MVA-GFP produced a lower level of GFP fluorescence than WR-GFP in the infected monocytes. Those differences could be the result of a lower expression level, or a delay in the course of infection, by the MVA strain.

In addition to increased GFP expression levels, WR-GFP was also more efficient than MVA-GFP for the infection of CD19^+ ^B lymphocytes (7.1% *vs *3.5%) and CD56^+^/CD16^+ ^NK cells(7.6% *vs *4%).

### Infection with thymidine kinase-deficient viruses

As stated above, we constructed recombinant viruses from both WR and MVA by insertion of GFP into the TK locus. Infection of different populations in human PBLs with these viruses was again monitored in paralell using specific antibodies (Fig. 2). Infection of PBLs with WR-TK(-) virus resulted in similar percentages of infected CD14 and CD56/CD16 positive cells, although a slight decrease of infection rates was noted in CD19 cells. The level of GFP fluorescence in CD14-positive cells (monocytes) (and to a lesser extent, in all the WR-TK(-) infected lymphoid subsets) was markedly reduced with respect to the WR-GFP virus.

## Discussion

Detection of cells infected by GFP-expressing vaccinia viruses provide a fast and sensitive method to measure virus infection [6]. In this report, we have taken advantage of this approach to measure infection in freshly prepared human PBLs. In combination with cell-type specific fluorescent antibodies, we have been able to study the rate of infection in different cell subset within the PBL population.

It is to note that the approach used in this work only allows the detection of viral gene expression derived from the infection, but does not address whether the infection results in the production of progeny virus. Early reports indicated that vaccinia virus cause cythopathic effect in human leukocytes, although only replicated in mitogen-stimulated cell populations, indicating that active cell replication is required for virus replication [7,8]. In this respect, it has also been reported that vaccinia infection of dendritic cells and monocytes/macrophages is abortive [9-11], and that dendritic cells and macrophages die by apoptosis upon infection [9,12-14]. Less clear is the case of transformed B lymphocyte cell lines, where virus infection has been described to be productive [9] and abortive [15] in different cell lines.

Our results point to a significant preference of vaccinia virus for certain cell types. In particular, monocytes were the most susceptible cells, followed by B cells and NK cells. In contrast, T cells were infected at very low rates. These observations are in broad agreement with previous studies, where different infection rates have been noted between monocytes and lymphocytes [16] and between B and T lymphocytes [17]. In our analysis, we have detected different rates of virus infection of different cells but at this point we cannot relate the differences in infection to differential virus binding, internalization or gene expression in different PBL cell lineages. In any event, the consequences of virus tropism in the pathogenicity of poxviruses remains to be further investigated.

Comparison of the patterns obtained with the two virus strains and their TK(-) mutants indicate that both the virus strain and the TK phenotype may determine the amount of gene expression, as was revealed by the intensity of GFP fluorescence in infected monocytes. In addition, the ability of the virus to infect certain cell types (CD19) seems to be affected to a certain extent by disruption of the TK gene. While this may be derived from our inability to detect those infected cells because of decreased gene expression, we cannot rule out a more direct requirement of TK activity in those cells.

MVA vaccinia virus strain has elicited much interest recently because of its safety record. Because clinical complications and side effects of smallpox vaccination are a critical issue in the event of mass vaccination, understanding the basis of MVA attenuation may lead to the development of better vaccine vectors. In this study, a number of differences were noted between the rates of infection obtained with WR and MVA virus strains. While both viruses were able to infect the monocyte population, WR infected B cells and the NK population (CD56, CD16 positive cells) more efficiently than MVA. Whether these observations have implications on the pathogenicity or immunogenicity of MVA will require further studies.

The fact that both WR and MVA showed a strong preference for certain cell populations indicate that, in addition to host range genes, there are other factors that might influence the infection rate of PBL cells. Those might include a variety of such as the accessibility and amount of receptors, ability to internalize the virus, and the metabolic state of the cell.

## Conclusions

Monocytes (CD14+ cells) were the cells in the PBL population that showed a greater susceptibility to vaccinia virus infection, as measured by viral gene expression. On the other hand, T lymphocytes (CD3+ cells) were infected with low efficiency. An intermediate susceptibility was detected in B lymphocytes (CD19+ cells) and NK (CD56+ cells). Both the use of a highly attenuated virus strain (MVA) or the disruption of the thymidine kinase gene lead to decreased gene expression in the infected cells. Those observations highlight the existence of a different degree of susceptibility to infection if PBL subpopulations, a fact that may have important implications in understanding virus pathogenicity and immunogenicity.

## Methods

### Cells, plasmids and virus

Vaccinia virus strain WR was grown and titrated in BSC-1 or CV-1 cells, grown in minimal essential medium (EMEM) supplemented with 5% fetal bovine serum (FBS) and 2 mM L-Glutamine. MVA virus and recombinants were grown in BHK-21 cells (ATCC CCL10) cultured in BHK medium containing 5% FBS, 3 g/ml tryptose phosphate broth and 0.01 M hepes. All cells were maintained in a 5% CO_2 _atmosphere at 37°C. Plasmid pRB21 [18] contains vaccinia virus gene F13L and flanking sequences, and a synthetic early/late promoter placed downstream of the P37 coding sequence.

### Construction of recombinant viruses expressing GFP

Plasmid pRBrsGFP, designed to mediate the insertion of the gene coding an enhanced version of the green fluorescent protein gene, rsGFP, (Quantum Biotechnologies, Inc.) was constructed as follows. rsGFP gene in plasmid pQBI25 was amplified using oligonucleotides GFP 5' (AATATAAATGGCTAGCAAAGGAGAAGAA) and GFPH3 (TTTAAAGCTTTACTAGTGGATCCTCAG), that include *NheI *and *HindIII *restriction sites, respectively. After digestion with *NheI *and *HindIII*, the gene was inserted into the corresponding sites in plasmid pRB21 [18], downstream of a synthetic vaccinia early/late promoter.

Plasmid prsGTK, containing the above GFP expression cassette located between recombination flanks for the TK locus, was obtained by cloning the rsGFP cassette from plasmid pRBrsGFP in plasmid pGPTK (Sanchez-Puig and Blasco, unpublished) after digestion with *XhoI *and *BamHI*.

Viruses WR-GFP and MVA-GFP were obtained by transfection of plasmid pRBrsGFP in cells infected with WR mutant vRB12 [19] or MVA, respectively. After plaquing of the progeny virus, GFP-positive virus plaques were identified by inspection in a Nikon TE-300 inverted fluorescence microscope, plaque-purified three times and amplified.

Recombinant virus VVrsGFPTK, was isolated after transfection of plasmid prsGTK in cells infected with virus WR. Recombinant virus plaques were isolated by plaquing on 143B TK(-) cells in the presence of 25 μg/ml bromodoxyuridine. GFP positive plaques were identified under the microscope, and plaque-purified three times before amplification.

Recombinant virus MVA-GFPTK was isolated by transfection of plasmid prsGTK in MVA-infected BHK-21 cells. GFP-positive virus were identified under the microscope, isolated by three consecutive rounds of plaque purification in BHK-21 cells and amplified in BHK-21 cell cultures.

Finally, virus recombinants were analyzed by Southern Blot, using digoxigenin-labelled GFP gene sequence as the probe. The analysis demonstrated that the recombinants contained the GFP expression cassette in the desired genome position and that they were stable, double recombinants.

### Isolation of human PBLs

Peripheral blood mononuclear cells from healthy subjects were obtained by density gradient centrifugation of heparinized blood on Ficoll-Paque (Pharmacia, Uppsala, Sweden). Cells obtained from the interface were washed three times in saline solution and then resuspended in complete medium (CM) consisting of RPMI 1640 (Gibco, Life Technologies, Germany) supplemented with 10% FBS (Gibco), 2 mM L-glutamine (ICN, USA), 100 U/ml each of penicillin and streptomycin (Laboratorios Normon, Spain). Viability of the isolated cells always exceeded 95% as determined by trypan blue exclusion.

Infection of human PBLs was performed as follows: 2 × 10^5 ^PBLs were infected with virus recombinants VV-rsGFP, VVrsGFPTK, MVA-GFP, and MVA-GFPTK, at 10 p.f.u./cell, in 0.7 ml of RPMI medium containing 2% FBS. After 1 h adsorption, cells were pelleted and resuspended in 2 ml of fresh RPMI medium containing 2% FBS. At different infection times, the cells were sedimented by low-speed centrifugation, resuspended in 100 μl FACS-FLOW, and labeled with the appropriate conjugated monoclonal antibodies (mAb) for flow cytometric analysis (FCM) (phycoerythrin, PE- peridinil chlorophyll protein, PerCP- and allophycocianin, APC-conjugated mAb directed against CD3, CD4, CD8, CD14 and CD16 were obtained from BD; mAb against CD19 and CD56 from Beckman Coulter).

Cells were incubated with the antibodies for 30 min at 4°C in the dark, washed twice with saline solution and finally resuspended in 200 μl Cytofix/Cytoperm (BD Pharmingen). Cells were analyzed in a FACSCalibur (BD Biosciences, San Diego, CA) and data were processed with Cell Quest software (BD).

## Competing interests

The author(s) declare that they have no competing interests.

## Authors' contributions

JMS carried out the isolation of virus recombinants and performed viral infections and participated in the drafting of the manuscript. LS and GR performed the preparation of PBLs, carried out the flow cytometry and elaborated the data. GR participated in the interpretation of the data and helped in the elaboration of the manuscript. RB conceived the study, designed the virus recombinant constructs, supervised the experimental work and drafted the manuscript. All authors read and approved the final manuscript.
